# Supplementary material for: Chronic social defeat stress induces meningeal neutrophilia via type I interferon signaling
Source: bioRxiv. 2024 Aug 31:2024.08.30.610447. Preprint. [Version 1] doi: 10.1101/2024.08.30.610447 (PMC11383661; doi:10.1101/2024.08.30.610447)
Supplement: Supplement 1 [file media-1.pdf]

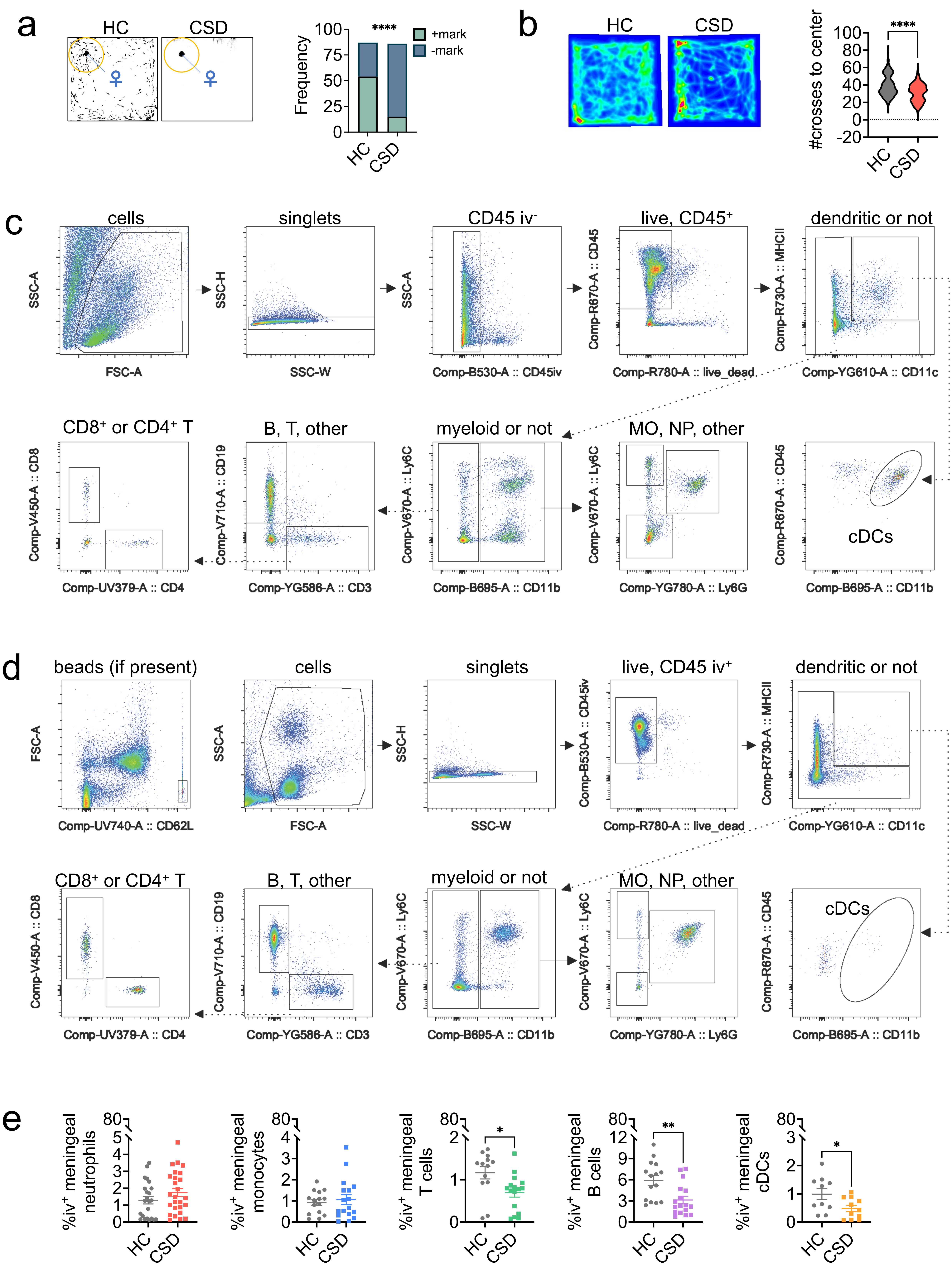
a
HC
CSD
Frequency
****
+mark
-mark
b
HC
CSD
#crosses to center
****
c
cells
singlets
CD45 iv⁻
live, CD45⁺
dendritic or not
SSC-A
FSC-A
SSC-H
SSC-W
Comp-B530-A :: CD45iv
Comp-R670-A :: CD45
Comp-R780-A :: live_dead
Comp-R730-A :: MHCII
Comp-YG610-A :: CD11c
CD8⁺ or CD4⁺ T
B, T, other
myeloid or not
MO, NP, other
cDCs
Comp-V450-A :: CD8
Comp-UV379-A :: CD4
Comp-V710-A :: CD19
Comp-YG586-A :: CD3
Comp-V670-A :: Ly6C
Comp-B695-A :: CD11b
Comp-YG780-A :: Ly6G
d
beads (if present)
cells
singlets
live, CD45 iv⁺
dendritic or not
FSC-A
Comp-UV740-A :: CD62L
Comp-B530-A :: CD45iv
e
%iv⁺ meningeal neutrophils
%iv⁺ meningeal monocytes
%iv⁺ meningeal T cells
%iv⁺ meningeal B cells
%iv⁺ meningeal cDCs
*
**

Figure S1

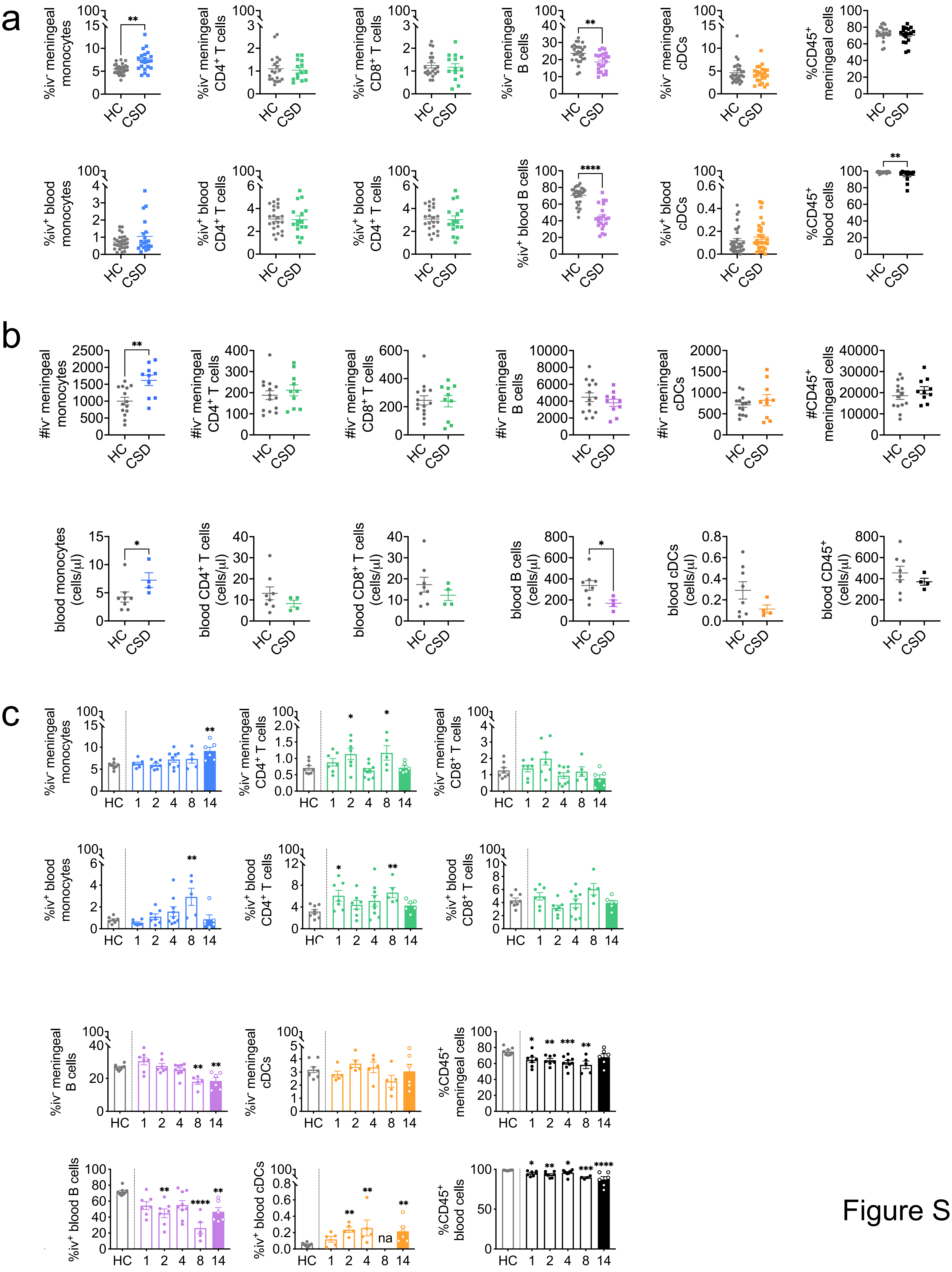
a
b
c
%iv- meningeal monocytes
%iv- meningeal CD4+ T cells
%iv- meningeal CD8+ T cells
%iv- meningeal B cells
%iv- meningeal cDCs
%CD45+ meningeal cells
%iv+ blood monocytes
%iv+ blood CD4+ T cells
%iv+ blood CD4+ T cells
%iv+ blood B cells
%iv+ blood cDCs
%CD45+ blood cells
#iv- meningeal monocytes
#iv- meningeal CD4+ T cells
#iv- meningeal CD8+ T cells
#iv- meningeal B cells
#iv- meningeal cDCs
#CD45+ meningeal cells
blood monocytes (cells/µl)
blood CD4+ T cells (cells/µl)
blood CD8+ T cells (cells/µl)
blood B cells (cells/µl)
blood cDCs (cells/µl)
blood CD45+ (cells/µl)
%iv+ blood CD8+ T cells
HC
CSD
1
2
4
8
14
na

Figure S2

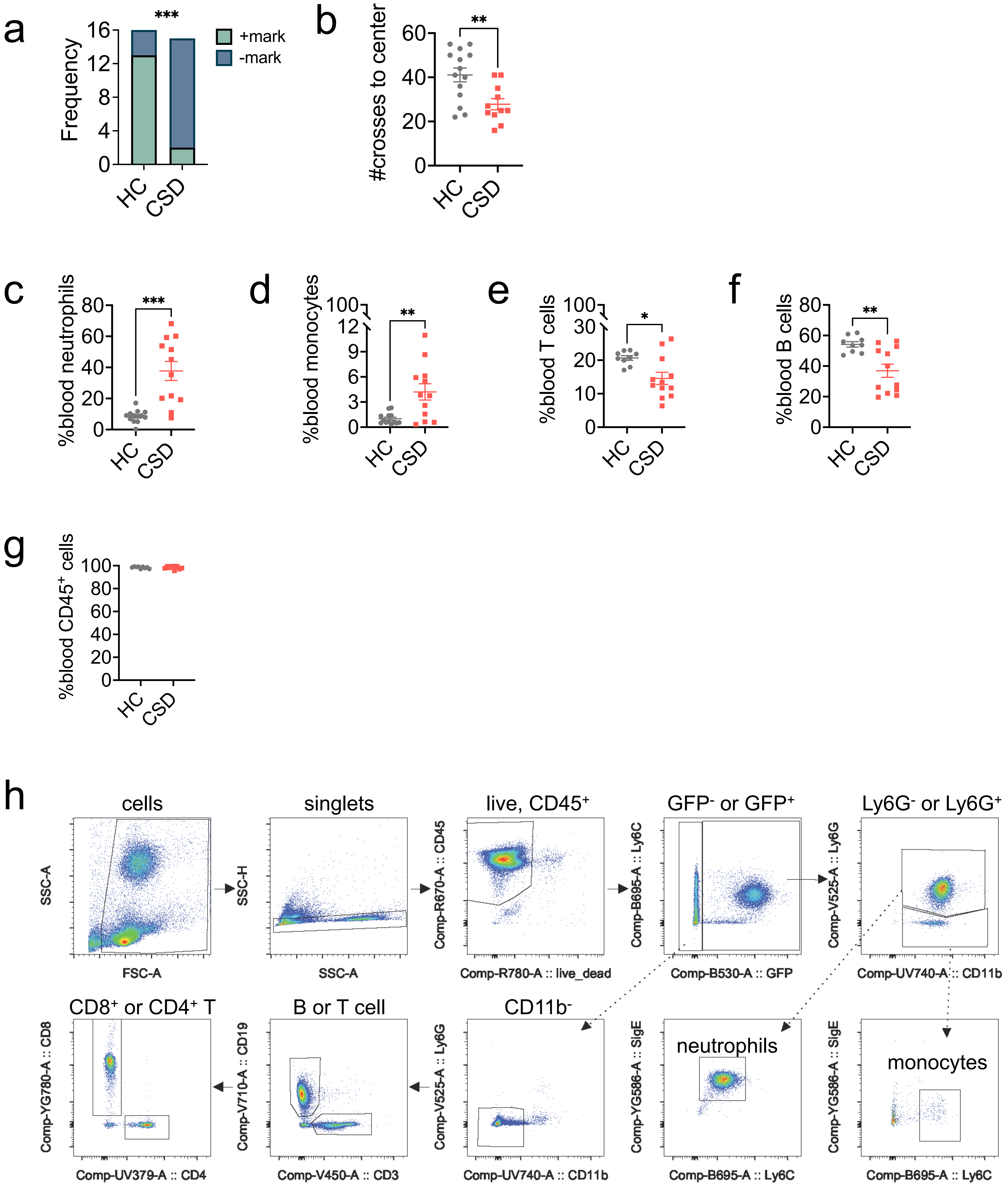
a
***
Frequency
+mark
-mark
HC
CSD
b
**
#crosses to center
c
***
%blood neutrophils
d
**
%blood monocytes
e
*
%blood T cells
f
**
%blood B cells
g
%blood CD45+ cells
h
cells
singlets
live, CD45+
GFP- or GFP+
Ly6G- or Ly6G+
SSC-A
FSC-A
SSC-H
Comp-R670-A :: CD45
Comp-R780-A :: live_dead
Comp-B695-A :: Ly6C
Comp-B530-A :: GFP
Comp-V525-A :: Ly6G
Comp-UV740-A :: CD11b
CD8+ or CD4+ T
B or T cell
CD11b-
neutrophils
monocytes
Comp-YG780-A :: CD8
Comp-UV379-A :: CD4
Comp-V710-A :: CD19
Comp-V450-A :: CD3
Comp-YG586-A :: SigE

Figure S3

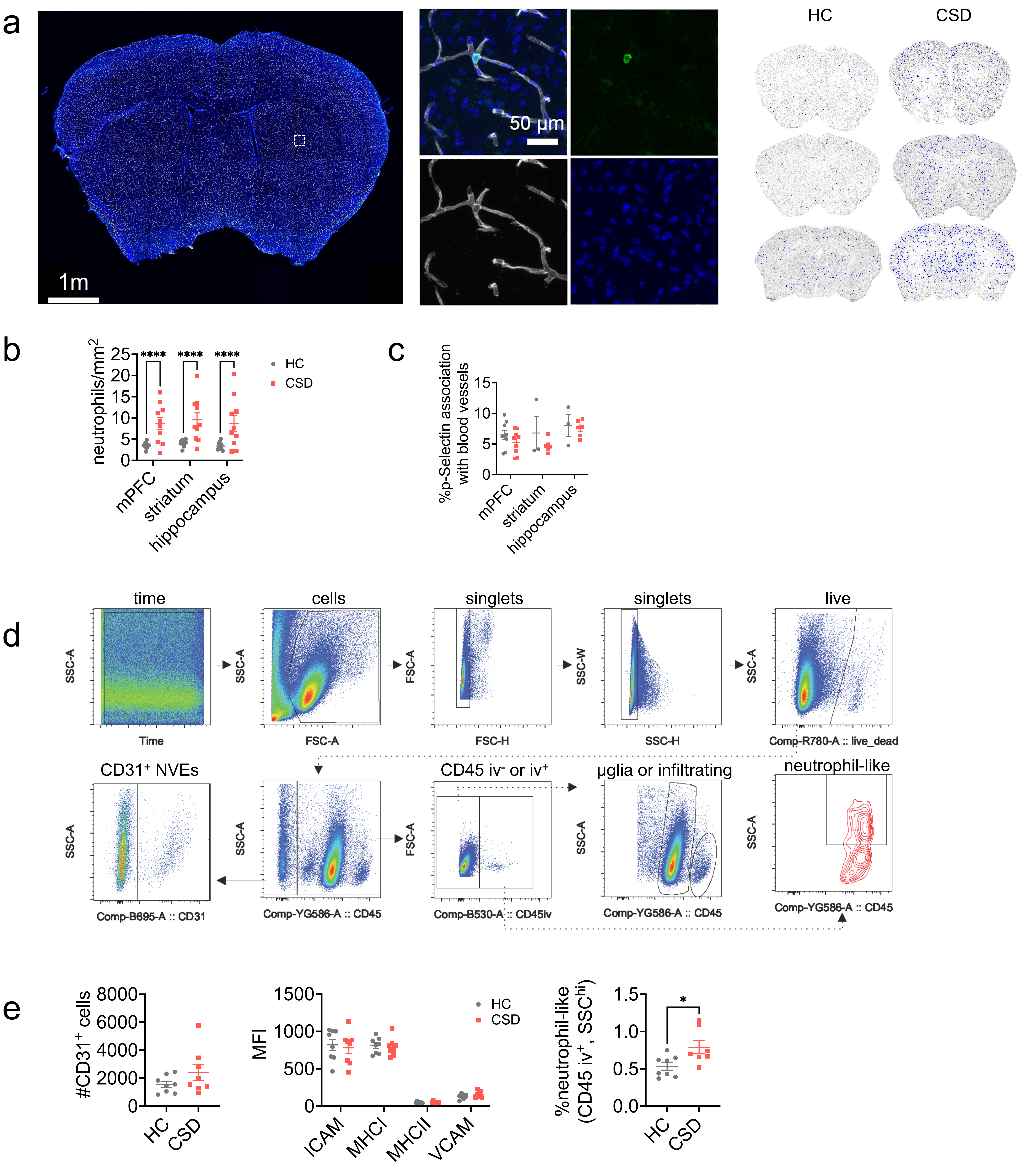
a
1m
50 µm
HC
CSD
b
neutrophils/mm2
****
HC
CSD
mPFC
striatum
hippocampus
c
%p-Selectin association with blood vessels
mPFC
striatum
hippocampus
d
time
cells
singlets
singlets
live
SSC-A
Time
FSC-A
FSC-H
SSC-W
SSC-H
Comp-R780-A :: live_dead
CD31+ NVEs
CD45 iv- or iv+
µglia or infiltrating
neutrophil-like
Comp-B695-A :: CD31
Comp-YG586-A :: CD45
Comp-B530-A :: CD45iv
Comp-YG586-A :: CD45
Comp-YG586-A :: CD45
e
#CD31+ cells
HC
CSD
MFI
ICAM
MHCI
MHCII
VCAM
%neutrophil-like (CD45 iv+, SSChi)
*

Figure S4

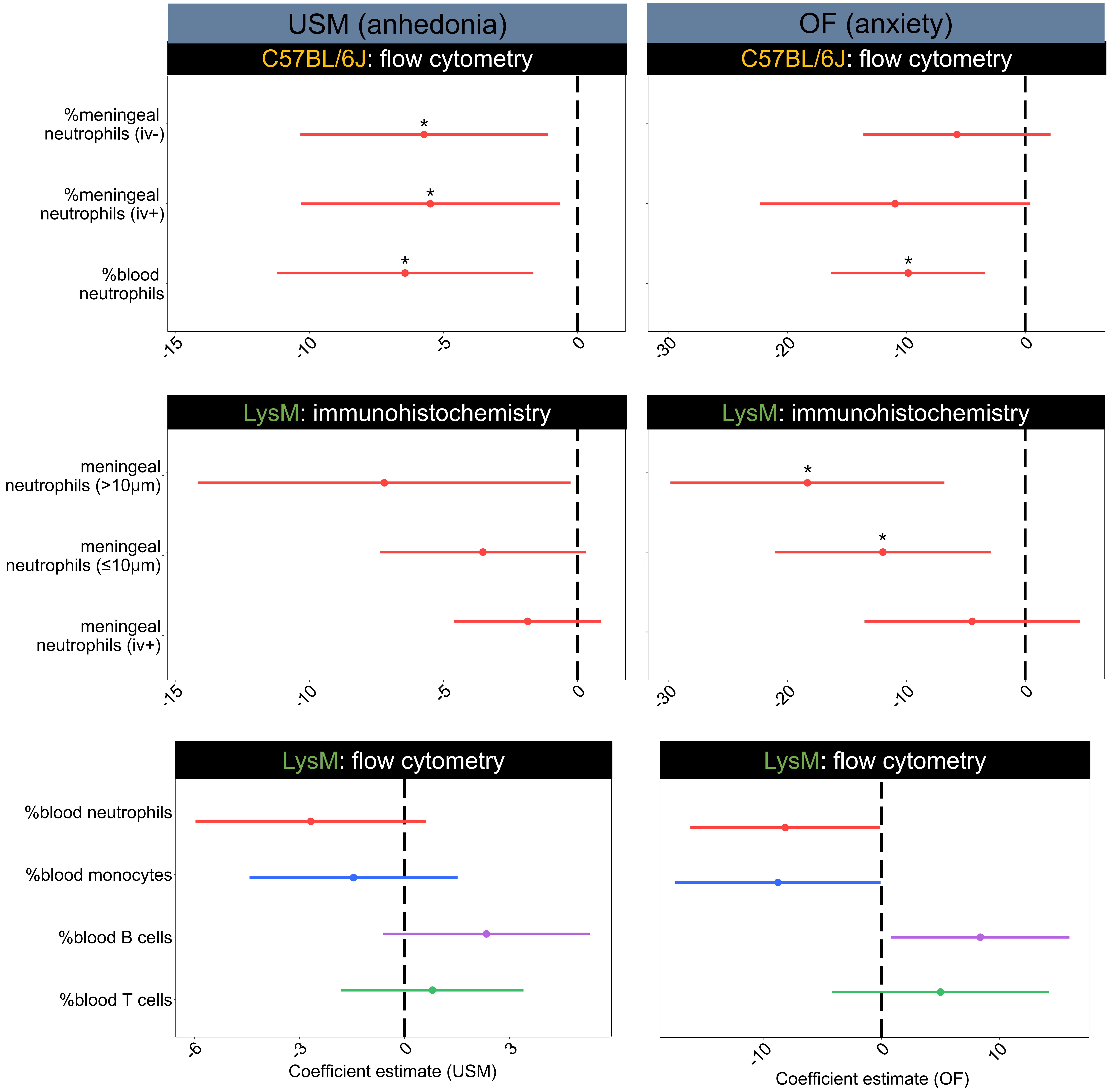
USM (anhedonia)
OF (anxiety)
C57BL/6J: flow cytometry
%meningeal neutrophils (iv-)
%meningeal neutrophils (iv+)
%blood neutrophils
LysM: immunohistochemistry
meningeal neutrophils (>10μm)
meningeal neutrophils (≤10μm)
meningeal neutrophils (iv+)
LysM: flow cytometry
%blood neutrophils
%blood monocytes
%blood B cells
%blood T cells
Coefficient estimate (USM)
Coefficient estimate (OF)

Figure S5

a Marker genes by % of cells expressing gene

Microglia	Preneutrophils	Neutrophils	Monocytes Ly6Chi	Monocytes NOS	Border-associated macrophage	Perivascular macrophage (Cd206)	cDCs	pDCs	Mast cells and others	Endothelial and others	Fibroblasts and other	Olfactory neurons	Pro B cells and other precursors	Pre B cells	Mature B cells 1	Mature B cells 2	T cells 1	T cells 2	NK cells	Erythrocytes
P2ry12	Gm10282	Cxcr2	Asf1b	Fn1	Ms4a7	Mrc1	Cd209a	Ccr9	Cdk6	Crip2	Col1a2	Elavl3	Myl4	Arl5c	Ms4a1	Fcmr	Cd3g	Cd3g	Klrb1c	Trim10
Gpr34	1700020L24Rik	Hdc	Cdca3	Ms4a8a	Cxcl16	Dab2	Tnip3	Cox6a2	Cpa3	Krt18	Rspo3	Fstl5	Mzb1	Fcrla	Tnfrsf13c	Gm31243	Il7r	Cd3d	Klre1	Gypa
Siglech	Orm1	Trem3	Ccna2	Clec4a1	Slamf9	Stab1	Clec4b1	Sh3bgr	Cst7	Flt1	Serpinh1	Gnal	Gm30211	Ebf1	Siglecg	H2-DMb2	Thy1	Thy1	Klrk1	Slc4a1
Tmem119	Ms4a3	Slc7a11	Racgap1	Clec4a3	C3ar1	Ms4a7	H2-DMb1	Cd300c	Gata2	Igfbp7	Aebp1	Scn9a	Ezh2	Tifa	Fcmr	Ms4a1	Lat	Ms4a4b	Klrd1	Alas2
Olfml3	Spc25	Mmp9	Mki67	Ccr2	H2-DMb1	Cbr2	Cbfa2t3	Siglech	Ms4a2	Timp3	Cald1	Sult1d1	Bcl7a	Vpreb3	Cd72	Bank1	Cd3d	Lck	Xcl1	Tspo2
Fcrls	Ccnb2	Fpr2	Smc2	Ms4a4c	Ctsc	Pf4	Slamf7	Klk1	Csrp3	Hspb1	Pcolce	Clgn	Smarca4	Pafah1b3	Spib	Cd79a	Skap1	Gimap4	Ncr1	Cldn13
Crybb1	Cdkn3	Pilra	Knstrn	F13a1	Tmem176a	Maf	Ccr2	Cd7	Srm	Ly6c1	Efemp1	Snap25	Sox4	Cd72	Cd79a	Ebf1	Cxcr6	Skap1	Txk	Rhd
Mafb	Inhba	Mxd1	Tacc3	AI839979	Lgmn	Folr2	Ccnd1	Upb1	Sfxn1	Tcf4	Plpp3	Flrt1	Lockd	Siglecg	Ebf1	H2-Ob	Bcl11b	Cd8b1	Ctsw	Fech
Hpgds	Cdca8	Il1r2	Ccnb2	Mcub	Tmem176b	Gas6	Mgl2	Runx2	Rcl1	Krt8	Igfbp5	Ttll7	Igll1	Sox4	Vpreb3	Cd79b	H2-Q7	Gimap3	Cd7	Sox6
Ecscr	Mki67	Chil1	Spc24	Cd300a	Aif1	Lyve1	Pid1	P2ry14	Gnl3	Fxyd6	Itm2a	Pcolce2	Akap12	Cnp	Pax5	Gm8369	Ccnd2	Ctsw	Ms4a4b	Snca

b Marker genes by level of expression

Microglia	Preneutrophils	Neutrophils	Monocytes Ly6Chi	Monocytes NOS	Border-associated macrophage	Perivascular macrophage (Cd206)	cDCs	pDCs	Mast cells and others	Endothelial and others	Fibroblasts and other	Olfactory neurons	Pro B cells and other precursors	Pre B cells	Mature B cells 1	Mature B cells 2	T cells 1	T cells 2	NK cells	Erythrocytes
Cst3	Camp	S100a8	Pclaf	Lyz2	Cd74	Apoe	H2-DMa	Irf8	Rpl15	Crip2	Malat1	Calm1	Ptma	Vpreb3	Ly6d	Rpl18a	Rpl17	Cd3d	Nkg7	Hba-a1
Hexb	Trem3	Retnlg	Lgals1	S100a4	H2-Aa	C1qa	H2-DMb1	Rpl31	Rps12	Ly6a	Mgp	Omp	Ptprcap	Ebf1	Cd79a	Rps19	Rps14	Rps15a	AW112010	Hbb-bs
Lgmn	H2afz	Cxcr2	S100a10	Lgals3	H2-Eb1	Pf4	Rps11	Plac8	Cmtm7	Krt18	Plpp3	Gng13	Stmn1	Chchd10	Cd79b	Fcmr	Tpt1	Hcst	Ccl5	Hbb-bt
P2ry12	Ngp	Bmx	Ly6c2	Ifitm3	H2-Ab1	Selenop	Plbd1	Bst2	Rpl14	Hspb1	Serpinf1	Nsg1	Tubb5	Arl5c	Ms4a1	Rps27	Rplp1	Cd3g	Klrk1	Car2
Tmem119	Hmgb2	S100a9	Tmsb10	F13a1	Tmem176b	Ctsb	Gm2a	Rpl10	Srgn	Ly6c1	Id3	Stoml3	Hmgb1	Dnajc7	Siglecg	Ltb	Il7r	Rps16	Klrb1c	Gpx1
C1qc	Hmgn2	Cxcl2	Tuba1b	Ms4a6c	Aif1	Dab2	Rps9	Siglech	Ifitm1	Tm4sf1	Igfbp5	Map1b	H2afv	Tifa	Btg1	Rpl13	Rpl36a	B2m	Ncr1	Hba-a2
Ctss	Wfdc21	Gsr	Pycard	Napsa	Ctsh	Cd68	Tnip3	Cox6a2	Ctsg	Cmtm8	1500015O10Rik	Tuba1a	Mzb1	Xrcc6	Ifi30	H2-DMb2	Rpl19	Gimap4	Klrd1	Prdx2
Sparc	Pglyrp1	Slc7a11	Ppia	Fn1	Tmem176a	Maf	Naaa	Sec61b	Npm1	Flt1	Col1a2	Calm2	Smarca4	Pafah1b3	Cd37	Rps29	Rplp0	Rpl13a	Xcl1	Gypa
Selplg	Lcn2	Itgam	Ran	Psap	Slamf9	Cbr2	Cdkn1a	Rpl36al	Ier3	Arhgap31	Rbp1	Tshz2	Pgls	Rhoh	Tnfrsf13c	Gm31243	Rps5	Ms4a4b	Gzma	Cd24a
C1qb	Anxa1	B430306N03Rik	Crip1	Smpdl3a	Fth1	Ftl1	Cd209a	Tcf4	Cst7	Sparcl1	Klf9	Plekhb1	Pkig	Fam53b	Cd72	Bank1	Cd163l1	Rps13	Id2	Alas2

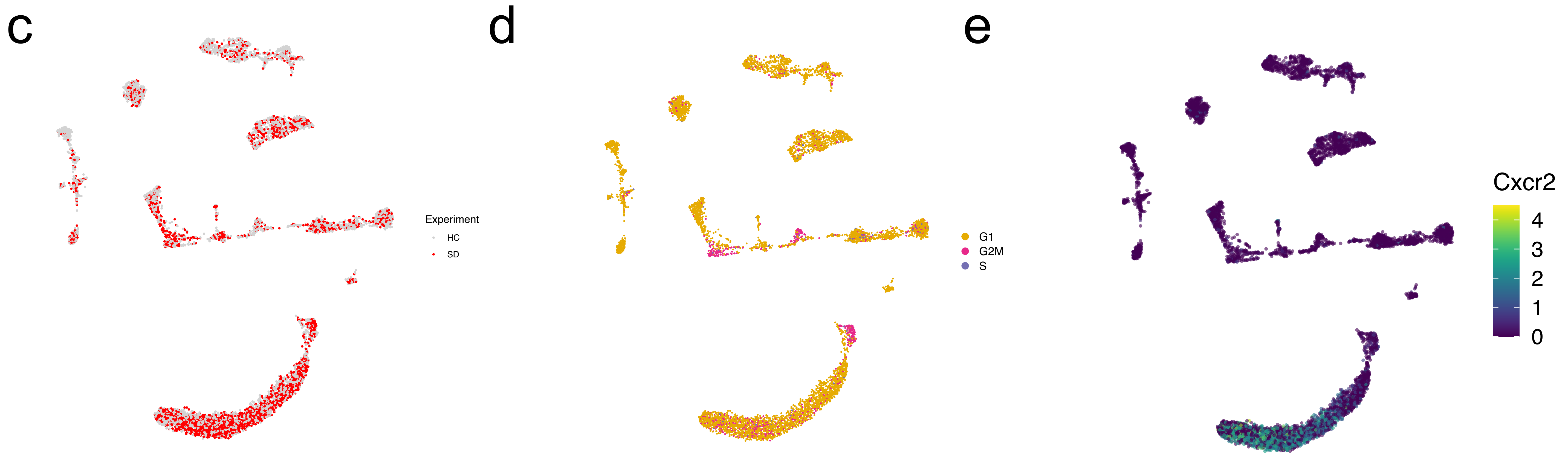

Figure S6

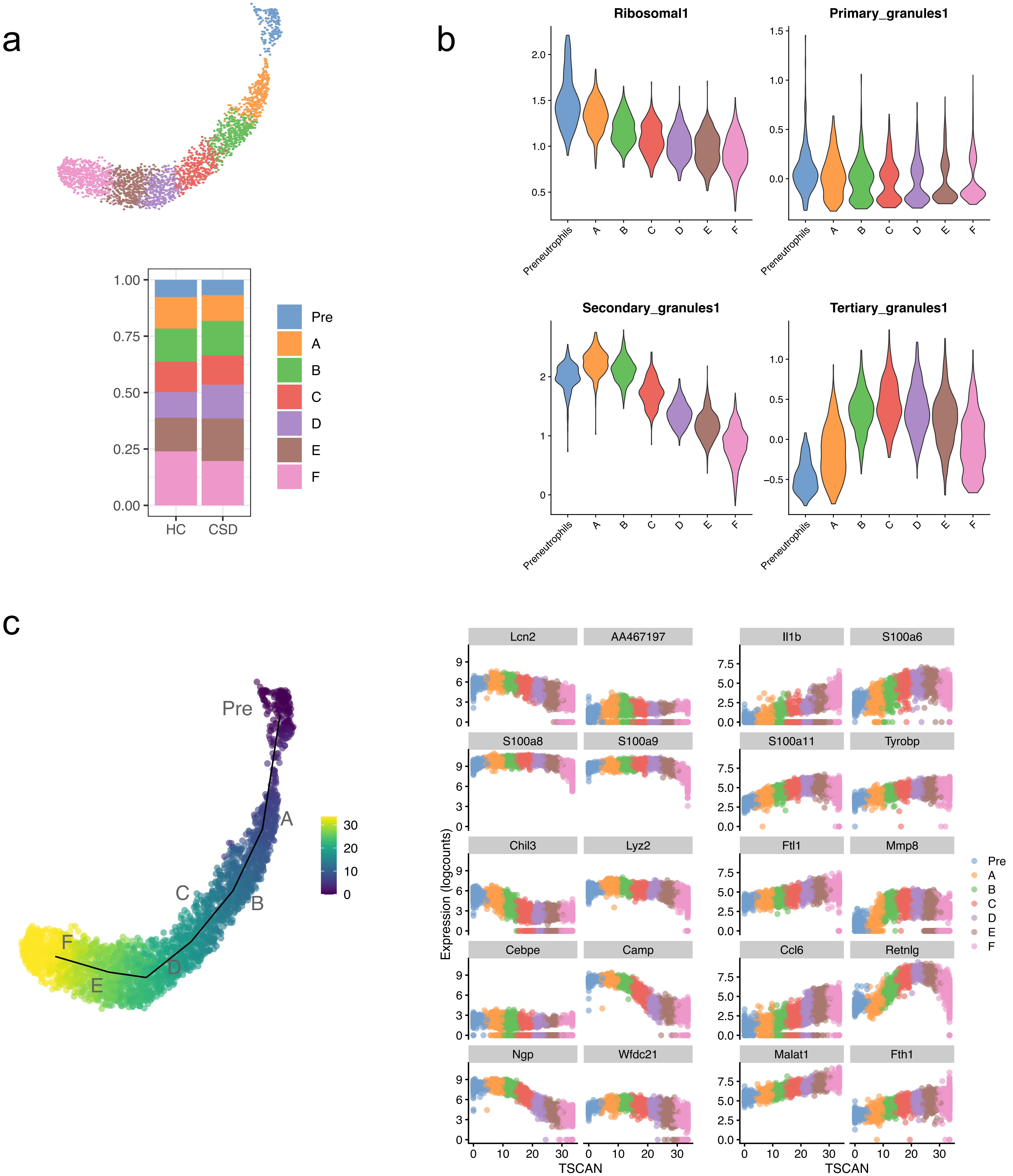

a
b
c
Pre
A
B
C
D
E
F
1.00
0.75
0.50
0.25
0.00
HC
CSD
Ribosomal1
Primary_granules1
Secondary_granules1
Tertiary_granules1
Preneutrophils
30
20
10
0
Lcn2
AA467197
Il1b
S100a6
S100a8
S100a9
S100a11
Tyrobp
Chil3
Lyz2
Ftl1
Mmp8
Cebpe
Camp
Ccl6
Retnlg
Ngp
Wfdc21
Malat1
Fth1
Expression (logcounts)
TSCAN

Figure S7

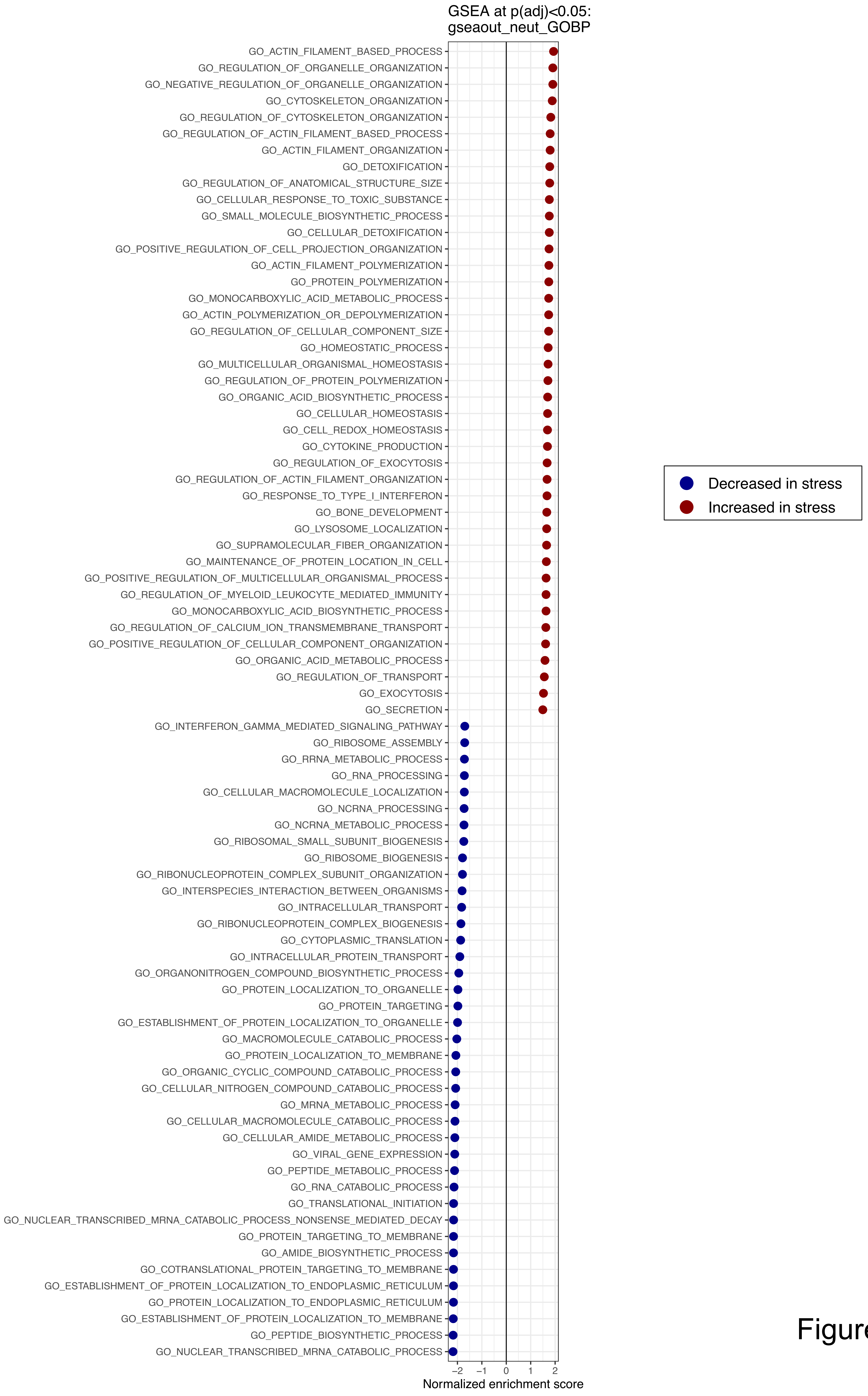
GSEA at p(adj)<0.05:
gseaout_neut_GOBP
GO_ACTIN_FILAMENT_BASED_PROCESS
GO_REGULATION_OF_ORGANELLE_ORGANIZATION
GO_NEGATIVE_REGULATION_OF_ORGANELLE_ORGANIZATION
GO_CYTOSKELETON_ORGANIZATION
GO_REGULATION_OF_CYTOSKELETON_ORGANIZATION
GO_REGULATION_OF_ACTIN_FILAMENT_BASED_PROCESS
GO_ACTIN_FILAMENT_ORGANIZATION
GO_DETOXIFICATION
GO_REGULATION_OF_ANATOMICAL_STRUCTURE_SIZE
GO_CELLULAR_RESPONSE_TO_TOXIC_SUBSTANCE
GO_SMALL_MOLECULE_BIOSYNTHETIC_PROCESS
GO_CELLULAR_DETOXIFICATION
GO_POSITIVE_REGULATION_OF_CELL_PROJECTION_ORGANIZATION
GO_ACTIN_FILAMENT_POLYMERIZATION
GO_PROTEIN_POLYMERIZATION
GO_MONOCARBOXYLIC_ACID_METABOLIC_PROCESS
GO_ACTIN_POLYMERIZATION_OR_DEPOLYMERIZATION
GO_REGULATION_OF_CELLULAR_COMPONENT_SIZE
GO_HOMEOSTATIC_PROCESS
GO_MULTICELLULAR_ORGANISMAL_HOMEOSTASIS
GO_REGULATION_OF_PROTEIN_POLYMERIZATION
GO_ORGANIC_ACID_BIOSYNTHETIC_PROCESS
GO_CELLULAR_HOMEOSTASIS
GO_CELL_REDOX_HOMEOSTASIS
GO_CYTOKINE_PRODUCTION
GO_REGULATION_OF_EXOCYTOSIS
GO_REGULATION_OF_ACTIN_FILAMENT_ORGANIZATION
GO_RESPONSE_TO_TYPE_I_INTERFERON
GO_BONE_DEVELOPMENT
GO_LYSOSOME_LOCALIZATION
GO_SUPRAMOLECULAR_FIBER_ORGANIZATION
GO_MAINTENANCE_OF_PROTEIN_LOCATION_IN_CELL
GO_POSITIVE_REGULATION_OF_MULTICELLULAR_ORGANISMAL_PROCESS
GO_REGULATION_OF_MYELOID_LEUKOCYTE_MEDIATED_IMMUNITY
GO_MONOCARBOXYLIC_ACID_BIOSYNTHETIC_PROCESS
GO_REGULATION_OF_CALCIUM_ION_TRANSMEMBRANE_TRANSPORT
GO_POSITIVE_REGULATION_OF_CELLULAR_COMPONENT_ORGANIZATION
GO_ORGANIC_ACID_METABOLIC_PROCESS
GO_REGULATION_OF_TRANSPORT
GO_EXOCYTOSIS
GO_SECRETION
GO_INTERFERON_GAMMA_MEDIATED_SIGNALING_PATHWAY
GO_RIBOSOME_ASSEMBLY
GO_RRNA_METABOLIC_PROCESS
GO_RNA_PROCESSING
GO_CELLULAR_MACROMOLECULE_LOCALIZATION
GO_NCRNA_PROCESSING
GO_NCRNA_METABOLIC_PROCESS
GO_RIBOSOMAL_SMALL_SUBUNIT_BIOGENESIS
GO_RIBOSOME_BIOGENESIS
GO_RIBONUCLEOPROTEIN_COMPLEX_SUBUNIT_ORGANIZATION
GO_INTERSPECIES_INTERACTION_BETWEEN_ORGANISMS
GO_INTRACELLULAR_TRANSPORT
GO_RIBONUCLEOPROTEIN_COMPLEX_BIOGENESIS
GO_CYTOPLASMIC_TRANSLATION
GO_INTRACELLULAR_PROTEIN_TRANSPORT
GO_ORGANONITROGEN_COMPOUND_BIOSYNTHETIC_PROCESS
GO_PROTEIN_LOCALIZATION_TO_ORGANELLE
GO_PROTEIN_TARGETING
GO_ESTABLISHMENT_OF_PROTEIN_LOCALIZATION_TO_ORGANELLE
GO_MACROMOLECULE_CATABOLIC_PROCESS
GO_PROTEIN_LOCALIZATION_TO_MEMBRANE
GO_ORGANIC_CYCLIC_COMPOUND_CATABOLIC_PROCESS
GO_CELLULAR_NITROGEN_COMPOUND_CATABOLIC_PROCESS
GO_MRNA_METABOLIC_PROCESS
GO_CELLULAR_MACROMOLECULE_CATABOLIC_PROCESS
GO_CELLULAR_AMIDE_METABOLIC_PROCESS
GO_VIRAL_GENE_EXPRESSION
GO_PEPTIDE_METABOLIC_PROCESS
GO_RNA_CATABOLIC_PROCESS
GO_TRANSLATIONAL_INITIATION
GO_NUCLEAR_TRANSCRIBED_MRNA_CATABOLIC_PROCESS_NONSENSE_MEDIATED_DECAY
GO_PROTEIN_TARGETING_TO_MEMBRANE
GO_AMIDE_BIOSYNTHETIC_PROCESS
GO_COTRANSLATIONAL_PROTEIN_TARGETING_TO_MEMBRANE
GO_ESTABLISHMENT_OF_PROTEIN_LOCALIZATION_TO_ENDOPLASMIC_RETICULUM
GO_PROTEIN_LOCALIZATION_TO_ENDOPLASMIC_RETICULUM
GO_ESTABLISHMENT_OF_PROTEIN_LOCALIZATION_TO_MEMBRANE
GO_PEPTIDE_BIOSYNTHETIC_PROCESS
GO_NUCLEAR_TRANSCRIBED_MRNA_CATABOLIC_PROCESS
−2
−1
0
1
2
Normalized enrichment score
Decreased in stress
Increased in stress

Figure S8

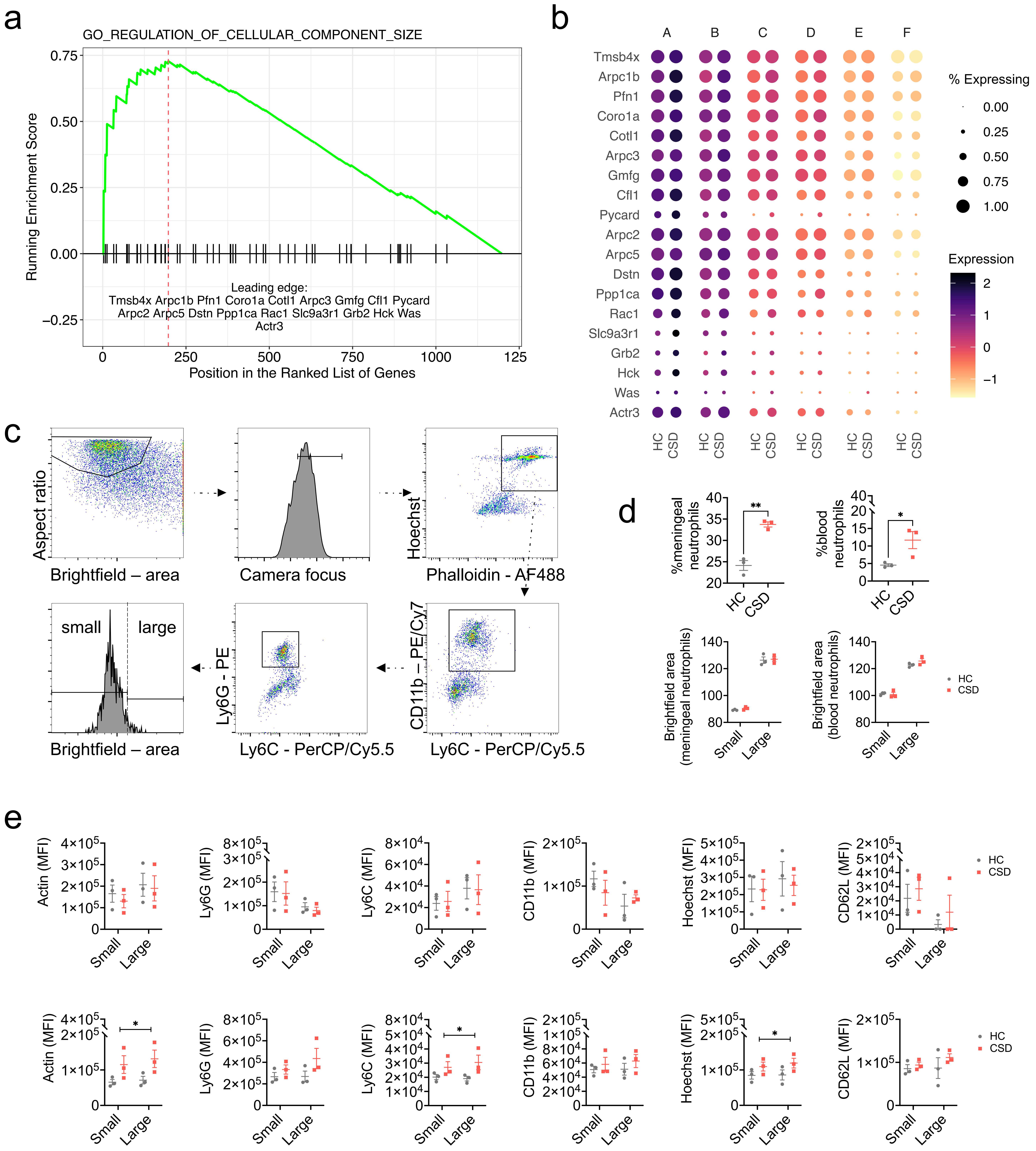

a
GO_REGULATION_OF_CELLULAR_COMPONENT_SIZE
Running Enrichment Score
Position in the Ranked List of Genes
Leading edge:
Tmsb4x Arpc1b Pfn1 Coro1a Cotl1 Arpc3 Gmfg Cfl1 Pycard
Arpc2 Arpc5 Dstn Ppp1ca Rac1 Slc9a3r1 Grb2 Hck Was
Actr3
b
A B C D E F
Tmsb4x
Arpc1b
Pfn1
Coro1a
Cotl1
Arpc3
Gmfg
Cfl1
Pycard
Arpc2
Arpc5
Dstn
Ppp1ca
Rac1
Slc9a3r1
Grb2
Hck
Was
Actr3
% Expressing
Expression
HC CSD
c
Aspect ratio
Brightfield – area
Camera focus
Hoechst
Phalloidin - AF488
small
large
Ly6G - PE
Ly6C - PerCP/Cy5.5
CD11b – PE/Cy7
d
%meningeal neutrophils
%blood neutrophils
Brightfield area (meningeal neutrophils)
Brightfield area (blood neutrophils)
Small
Large
HC
CSD
e
Actin (MFI)
Ly6G (MFI)
Ly6C (MFI)
CD11b (MFI)
Hoechst (MFI)
CD62L (MFI)

Figure S9

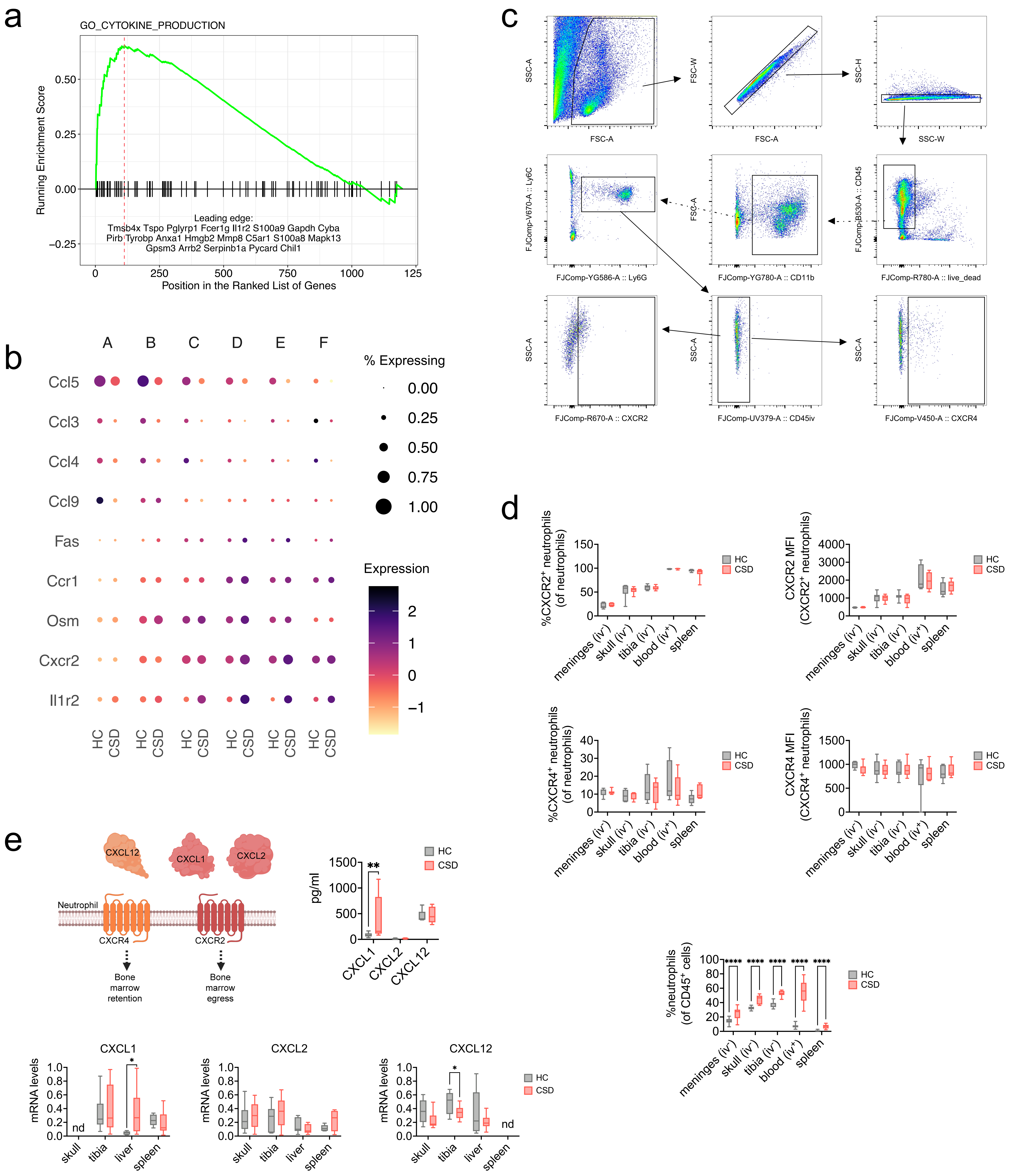
a
GO_CYTOKINE_PRODUCTION
Running Enrichment Score
Position in the Ranked List of Genes
Leading edge:
Tmsb4x Tspo Pglyrp1 Fcer1g Il1r2 S100a9 Gapdh Cyba
Pirb Tyrobp Anxa1 Hmgb2 Mmp8 C5ar1 S100a8 Mapk13
Gpsm3 Arrb2 Serpinb1a Pycard Chil1
b
A B C D E F
Ccl5 Ccl3 Ccl4 Ccl9 Fas Ccr1 Osm Cxcr2 Il1r2
HC CSD
% Expressing
Expression
c
SSC-A FSC-A FSC-W SSC-H SSC-W
FJComp-V670-A :: Ly6C
FJComp-YG586-A :: Ly6G
FJComp-YG780-A :: CD11b
FJComp-B530-A :: CD45
FJComp-R780-A :: live_dead
FJComp-R670-A :: CXCR2
FJComp-UV379-A :: CD45iv
FJComp-V450-A :: CXCR4
d
%CXCR2+ neutrophils (of neutrophils)
CXCR2 MFI (CXCR2+ neutrophils)
%CXCR4+ neutrophils (of neutrophils)
CXCR4 MFI (CXCR4+ neutrophils)
meninges (iv-) skull (iv-) tibia (iv-) blood (iv+) spleen
HC CSD
e
CXCL12 CXCL1 CXCL2
Neutrophil
CXCR4 CXCR2
Bone marrow retention
Bone marrow egress
pg/ml
CXCL1 CXCL2 CXCL12
%neutrophils (of CD45+ cells)
mRNA levels
skull tibia liver spleen
nd

Figure S10

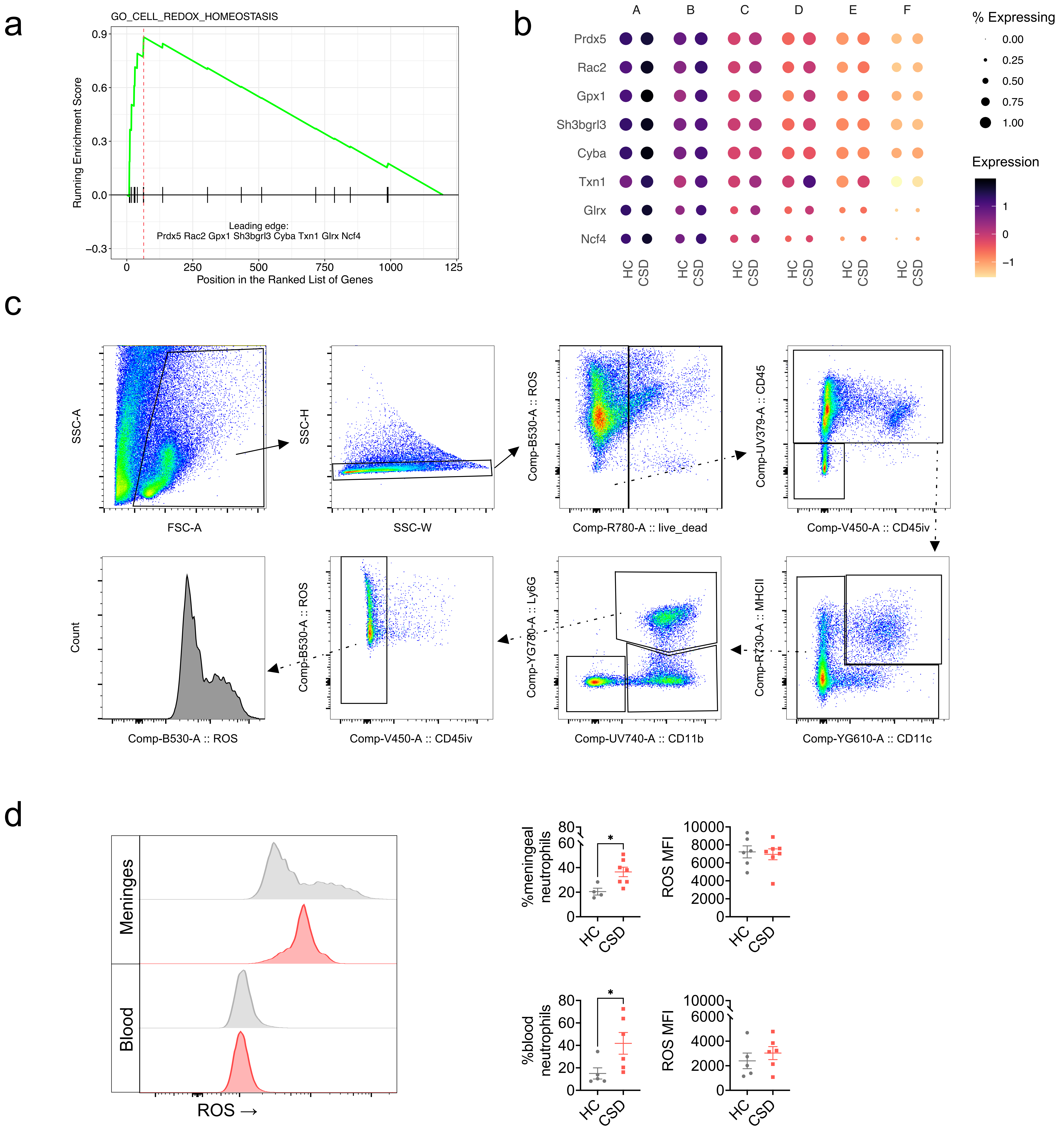

a
GO_CELL_REDOX_HOMEOSTASIS
Running Enrichment Score
Leading edge:
Prdx5 Rac2 Gpx1 Sh3bgrl3 Cyba Txn1 Glrx Ncf4
Position in the Ranked List of Genes
b
A B C D E F
Prdx5
Rac2
Gpx1
Sh3bgrl3
Cyba
Txn1
Glrx
Ncf4
HC CSD
% Expressing
0.00
0.25
0.50
0.75
1.00
Expression
c
SSC-A
FSC-A
SSC-H
SSC-W
Comp-B530-A :: ROS
Comp-R780-A :: live_dead
Comp-UV379-A :: CD45
Comp-V450-A :: CD45iv
Count
Comp-YG780-A :: Ly6G
Comp-UV740-A :: CD11b
Comp-R730-A :: MHCII
Comp-YG610-A :: CD11c
d
Meninges
Blood
ROS →
%meningeal neutrophils
%blood neutrophils
ROS MFI
HC
CSD

Figure S11

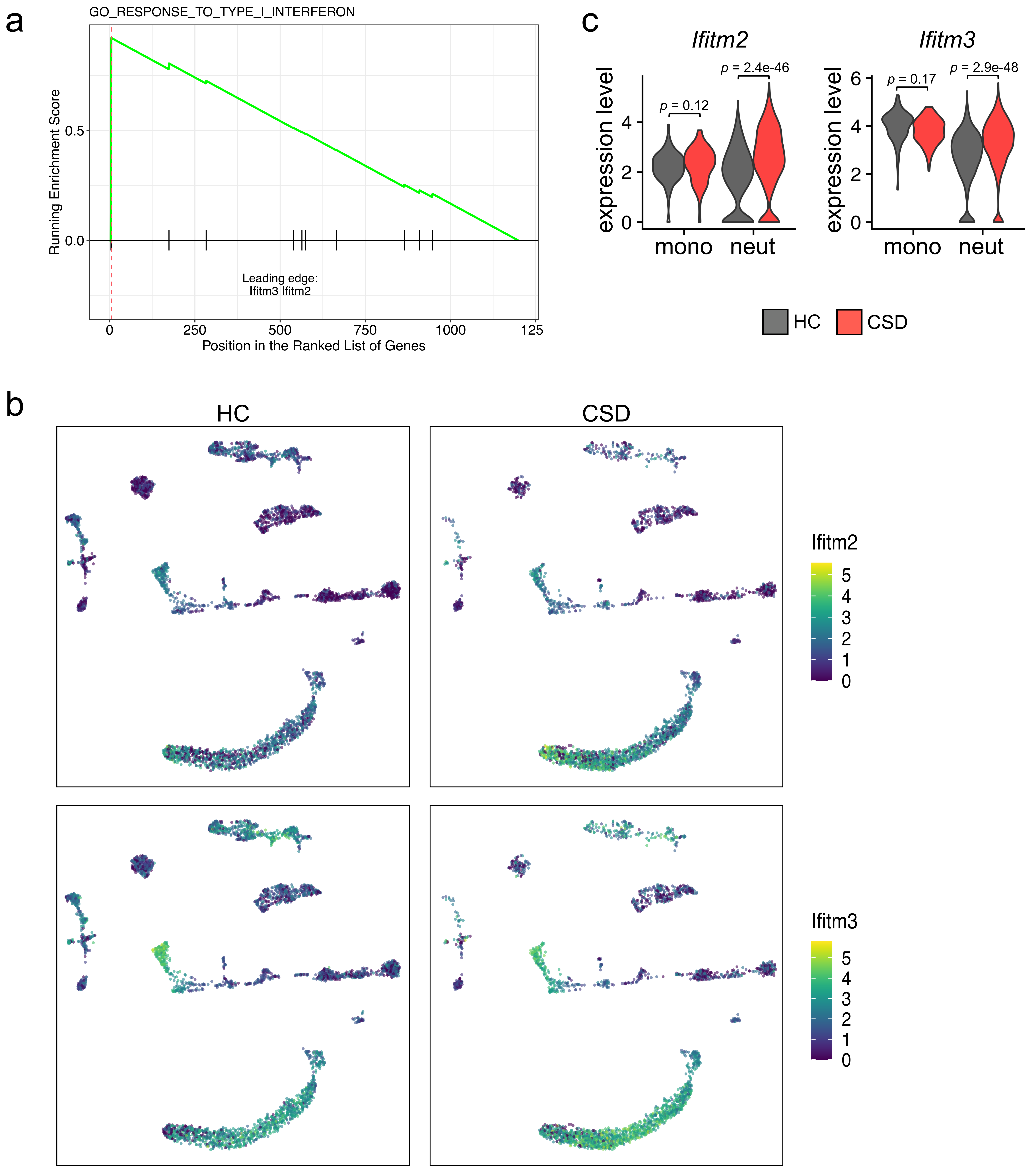

a
GO_RESPONSE_TO_TYPE_I_INTERFERON
Running Enrichment Score
0.5
0.0
Leading edge:
Ifitm3 Ifitm2
0
250
500
750
1000
125
Position in the Ranked List of Genes
c
Ifitm2
Ifitm3
expression level
p = 0.12
p = 2.4e-46
p = 0.17
p = 2.9e-48
mono
neut
HC
CSD
b
HC
CSD
Ifitm2
Ifitm3
5
4
3
2
1
0

Figure S12

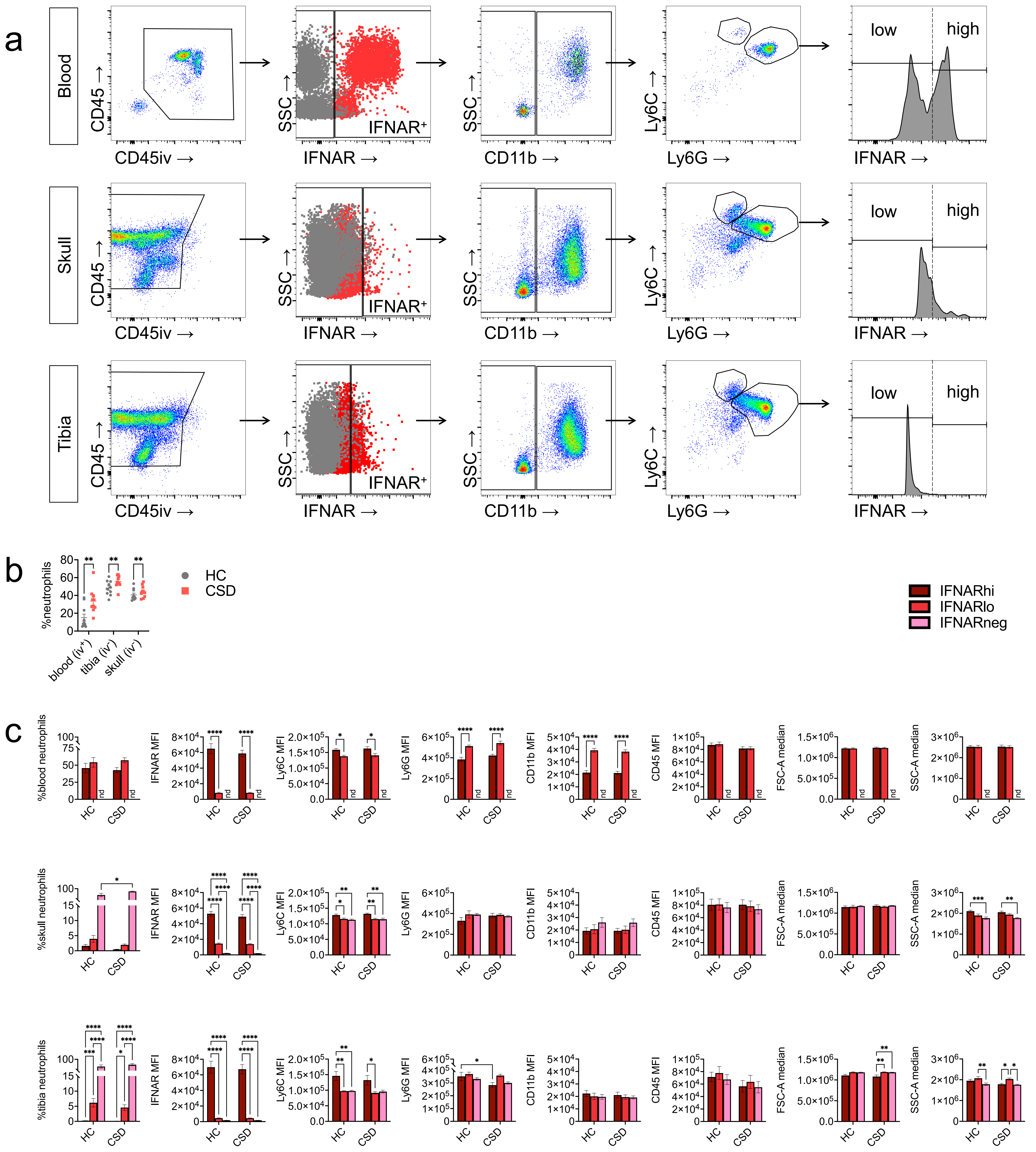
a
Blood
Skull
Tibia
CD45 →
CD45iv →
SSC →
IFNAR →
IFNAR+
CD11b →
Ly6C →
Ly6G →
low
high
b
%neutrophils
HC
CSD
blood (iv+)
tibia (iv-)
skull (iv-)
IFNARhi
IFNARlo
IFNARneg
c
%blood neutrophils
%skull neutrophils
%tibia neutrophils
IFNAR MFI
Ly6C MFI
Ly6G MFI
CD11b MFI
CD45 MFI
FSC-A median
SSC-A median
nd

Figure S13

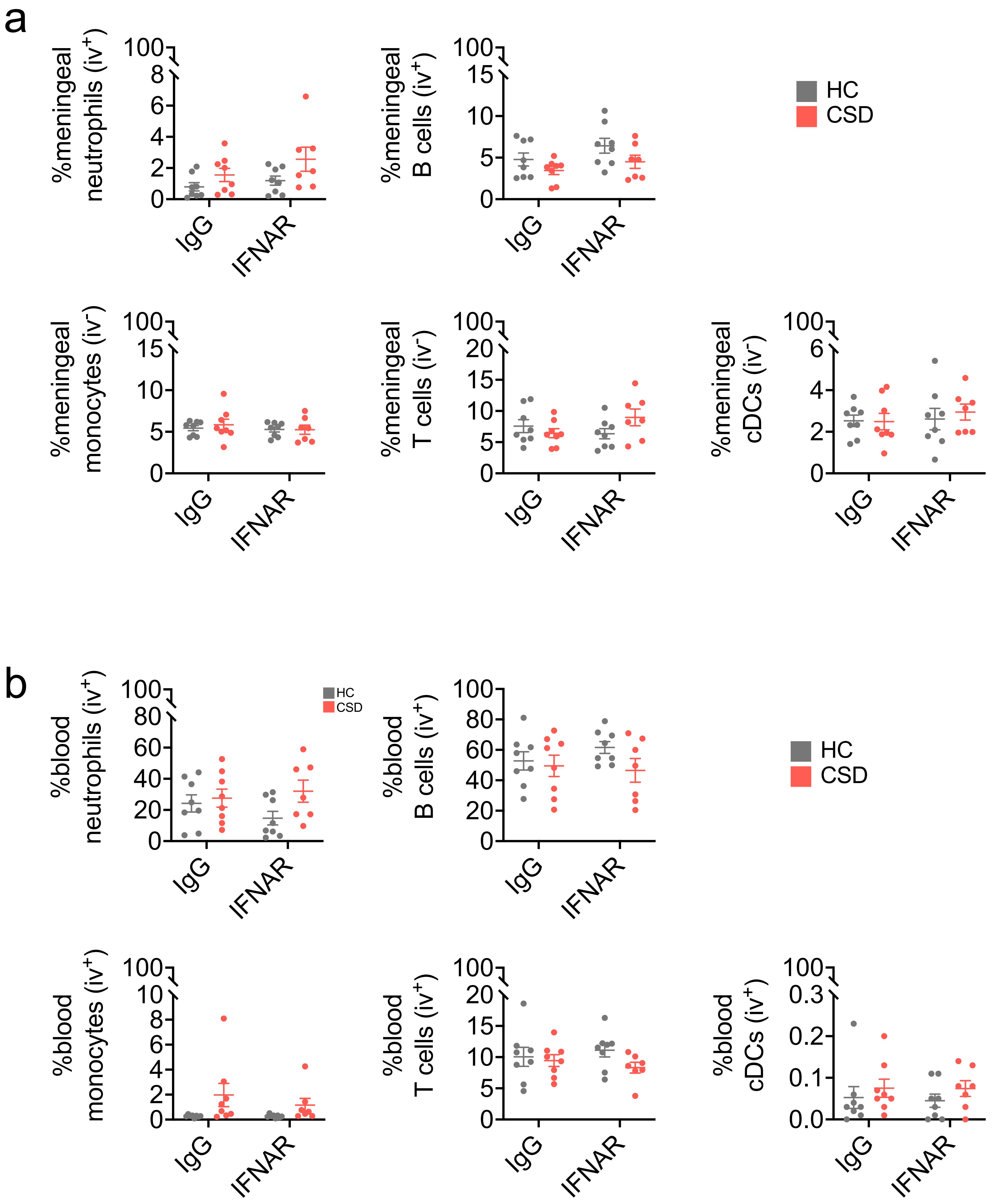
a
%meningeal neutrophils (iv+)
%meningeal B cells (iv+)
HC
CSD
%meningeal monocytes (iv-)
%meningeal T cells (iv-)
%meningeal cDCs (iv-)
IgG
IFNAR
b
%blood neutrophils (iv+)
%blood B cells (iv+)
%blood monocytes (iv+)
%blood T cells (iv+)
%blood cDCs (iv+)

Figure S14

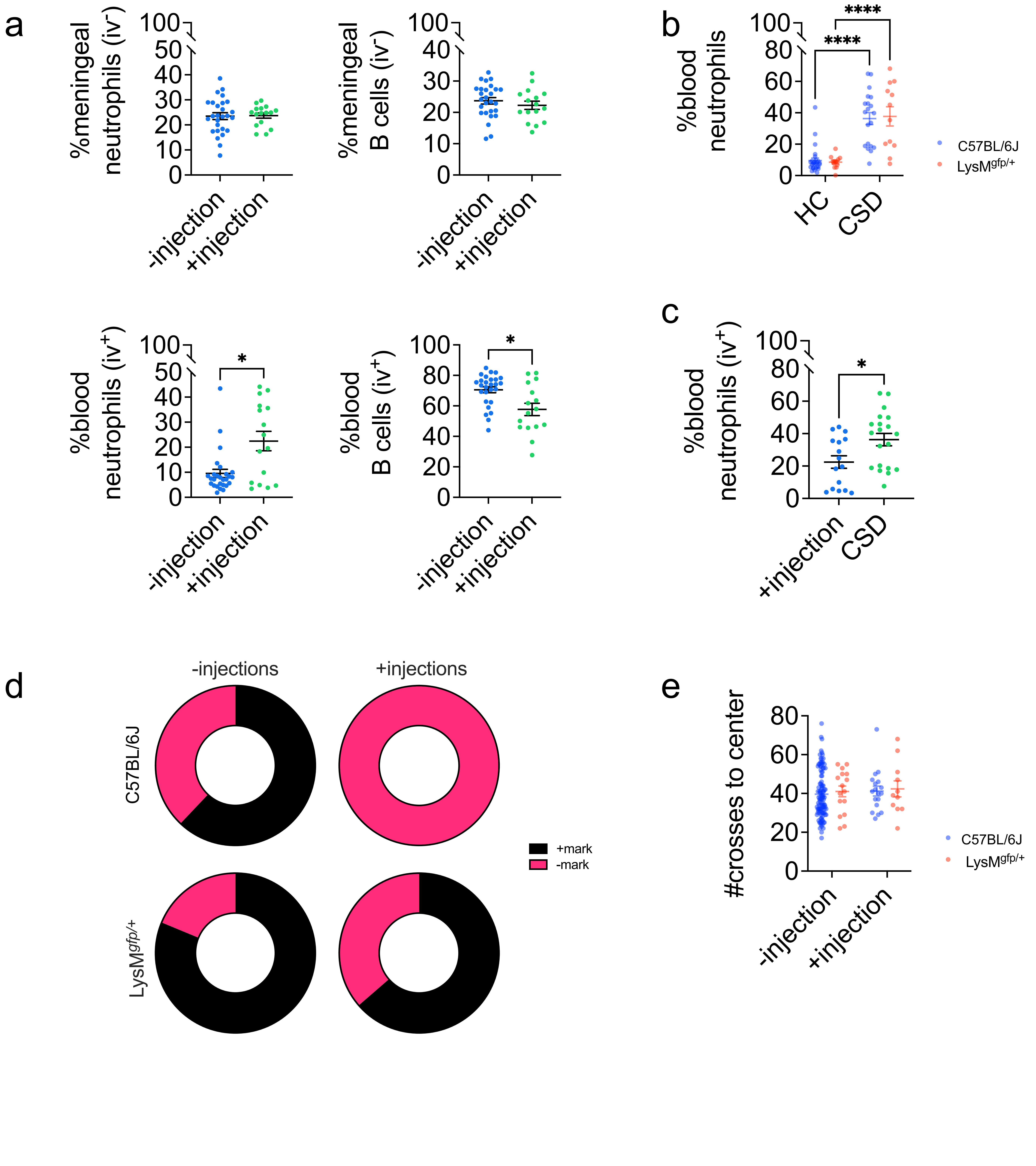

a
%meningeal neutrophils (iv⁻)
-injection
+injection
%meningeal B cells (iv⁻)
%blood neutrophils (iv⁺)
*
%blood B cells (iv⁺)
b
%blood neutrophils
****
HC
CSD
C57BL/6J
LysMgfp/+
c
+injection
d
-injections
+injections
LysMgfp/+
+mark
-mark
e
#crosses to center

Figure S15
